# Effect of Gibberellic Acid on Growing-Point Development of Non-Vernalized Wheat Plants under Long-Day Conditions

**DOI:** 10.3390/plants9121735

**Published:** 2020-12-09

**Authors:** Milan Skalicky, Jan Kubes, Pavla Vachova, Shokoofeh Hajihashemi, Jaroslava Martinkova, Vaclav Hejnak

**Affiliations:** 1 Department of Botany and Plant Physiology, Faculty of Agrobiology, Food and Natural Resources, Czech University of Life Sciences, 16500 Prague, Czech Republic; kubes@af.czu.cz (J.K.); vachovap@af.czu.cz (P.V.); martinkova@spika.cz (J.M.); hejnak@af.czu.cz (V.H.); 2 Plant Biology Department, Faculty of Science, Behbahan Khatam Alanbia University of Technology, Khuzestan 63616-47189, Iran; hajihashemi@bkatu.ac.ir

**Keywords:** double ridge, GA_3_, growing point, chlorophyll fluorescence

## Abstract

The goal of this study was to determine whether the application of gibberellic acid (GA_3_) to seeds of common wheat varieties with different vernalization and photoperiod requirements affects the transition from vegetative to generative stage. Three varieties of wheat with different photoperiod sensitivities and vernalization were selected for the experiment—the winter varieties, Mironovskaya and Bezostaya, and the spring variety, Sirael. Seeds were treated with different concentrations of GA_3_ and plants were grown under long-day conditions with monitoring of their photosynthetic activity (F_v_/F_m_, P_n_, E, g_s_). We monitored the activity of the photosynthetic apparatus by checking the plants to see if they were growing properly. The phenological stages of the wheat species were checked for indications of a transition from the vegetative to the generative stage. Selected concentrations of GA_3_ had no effect on the compensation of the vernalization process (transition to the generative phase). Chlorophyll fluorescence was one of the factors for monitoring stress. The variety, Bezostaya, is similar to the spring variety, Sirael, in its trends and values. The growth conditions of Bezostaya and Sirael were not affected by the activity of the photosynthetic apparatus. The development of growing points in winter varieties occurred at the prolonged single ridge stage. The spring variety reached the stage of head emergence after sixty days of growth (changes to the flowering phase did not appear in winter wheat). Application of GA_3_ to the seeds had no effect on the transition of the growing point to the double-ridge generative stage. The present study highlights the priming effect of GA_3_ on seeds of common wheat varieties with different vernalization and photoperiod requirements as it affected the transition from vegetative to generative stage.

## 1. Introduction

In wheat (*Triticum aestivum* L.), flowering is regulated genetically but is also influenced by environmental factors such as photoperiod and temperature [1]. The adaptability of wheat to a wide range of environments is favored by allelic diversity in genes regulating growth habit (*VRN*) and photoperiod response (*PPD*) [2,3]. Wheat is primarily a long-day (LD) plant that requires a photoperiod of fourteen hours or more [4]. The major environmental signal modulating flowering time is the photoperiod, the variation in day length during the growing season [5]. Natural variations in photoperiod response are mainly determined by allelic differences in the *PPD1* gene [3,6] and the photoperiod requirement may differ at different stages of development. Plants generally have different photoperiod requirements prior to flowering and after flowering [7]. In addition to a specific photoperiod, wheat requires a period of low temperature (vernalization) for flowering to occur [8]. Genetic differences in the growth type are determined by the *VRN1*, *VRN2* and *VRN3* genes, each having two or more alleles [9]. For varieties to manifest spring growth, the dominant allele, *Vrn*, must be present in the genome, and dominant alleles are inhibitors of the vernalization requirement. In winter varieties, all three *vrn* loci occur in recessive form [10]. Vernalization requirements are strong, but not identical in all sensitive genotypes. The vernalization process can only take place in cells that are dividing [11,12]. Protein synthesis is activated in germinating wheat embryos after thirty minutes of water imbibition [13], but active DNA synthesis cannot be observed in such embryos until fifteen hours after germination [14].

Gibberellins (GAs) are an important group of diterpene plant hormones that control diverse aspects of growth and development of plants from germination to flowering to seed formation [15,16,17]. According to [18], GA_3_ increases the number of wheat plants able to reach the threshold of inductive generative development, but the timing of flowering is not affected. Studies of grasses [19] indicated that the application of GAs can replace the requirement for a long day and promote flowering. After a spray application of GA_3_, *Sorghum bicolor* created flower bases faster than control plants [20], but the chlorophyll content was not affected by GA_3_ treatment. The functionality of the photosynthetic apparatus in terms of the PSII level can be evaluated by measuring the fluorescence of chlorophyll *a*. Measuring chlorophyll fluorescence is a non-invasive method for determining PSII activity used in studying plant physiology [21] and in evaluating responses of plants to environmental stress [22].

The ratio of variable to maximum fluorescence (F_v_/F_m_), the potential photochemical effectiveness of electron transport in the PSII, is one of the most useful measurements for evaluating photosynthetic activity [23]. F_v_/F_m_ is a general indicator of decreased function due to damage of the reactive center of photosystem II. It is frequently used to assess the quality of growth in wheat [24]. PSII activity is also quantitatively hereditary and sensitive to environmental changes [25]. Measurements of F_v_/F_m_ are rapid and sensitive indicators of changes in photosynthesis as well as changes in the overall physiological condition of the plant caused by environmental stressors [26,27], such as water stress [28], osmotic stress and excessive irradiance [29]. Measurement of chlorophyll *a* fluorescence was also used to determine if anthocyanins could protect plants from photoinhibition damage to PS II [30]. It was used to evaluate the effect of potassium on PS II [31], and for monitoring low temperature damage to the photosynthetic apparatus [32]. The method is accurate, reliable, and widely applicable for evaluating the condition of the photosynthetic apparatus as indicated by the level of photosynthesis.

This investigation was based on the observation that treatment of dwarf varieties of rye with GAs increased the activity of α-amylase in the germinating caryopses [33]. Treating seeds with GA_3_ increased α-amylase expression through binding of a transcription factor to the gene, which activated it [34]. Dwarf rye varieties are able to synthesize GA_3_ under certain conditions, and the normal growth of these varieties can be restored by exposure to exogenous GA_3_ [35]. GA-deficient mutants require exogenous GA for germinating seeds to complete the germination process [36]. Application of GA_3_ to germinating seeds might similarly help in eliminating the vernalization requirement. A GA_3_ concentration of 50 mg L^−1^ had a positive effect on germination and seed development, but 200 mg L^−1^ was inhibitory [37].

The goal of this study was to determine whether and how the exogenous application of GA_3_ to seeds of common wheat varieties with different vernalization and photoperiod requirements affected the transition from vegetative to generative stage. Simultaneously, the effect of long-day was monitored in two varieties of winter wheat with different photoperiod-sensitivities. Exposure to long day irradiance is atypical for winter wheat varieties in the early stages of their development.

## 2. Results and Discussion

**Figure 1:**
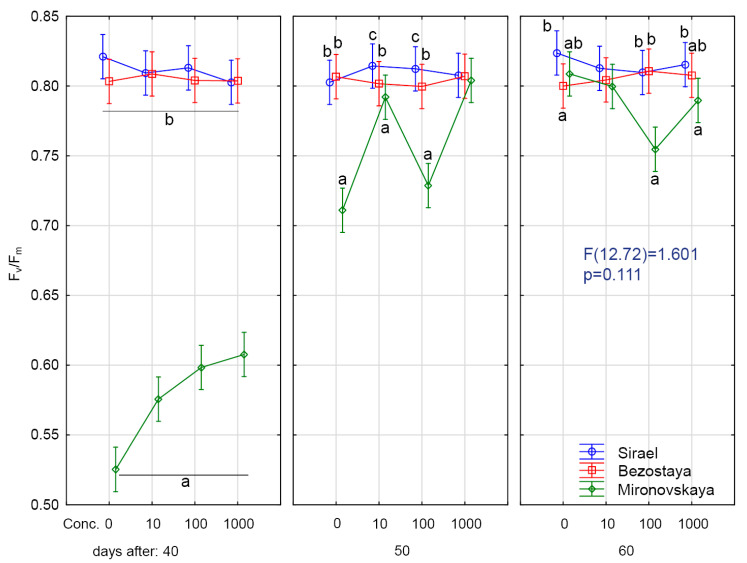
Optimal quantum yield (F_v_/F_m_) after GA_3_ treatment. Variety: Mironovskaya (M), Bezostaya (B) and Sirael (S). Concentrations of GA_3_: 0, 10, 100 a 1000 µL L^−1^; measured on 40 days, 50 days and 60 days of growth. Tukey HSD test, *α* = 0.05. Treatments with the same sign did not differ significantly at *p* < 0.05.

**Figure 2:**
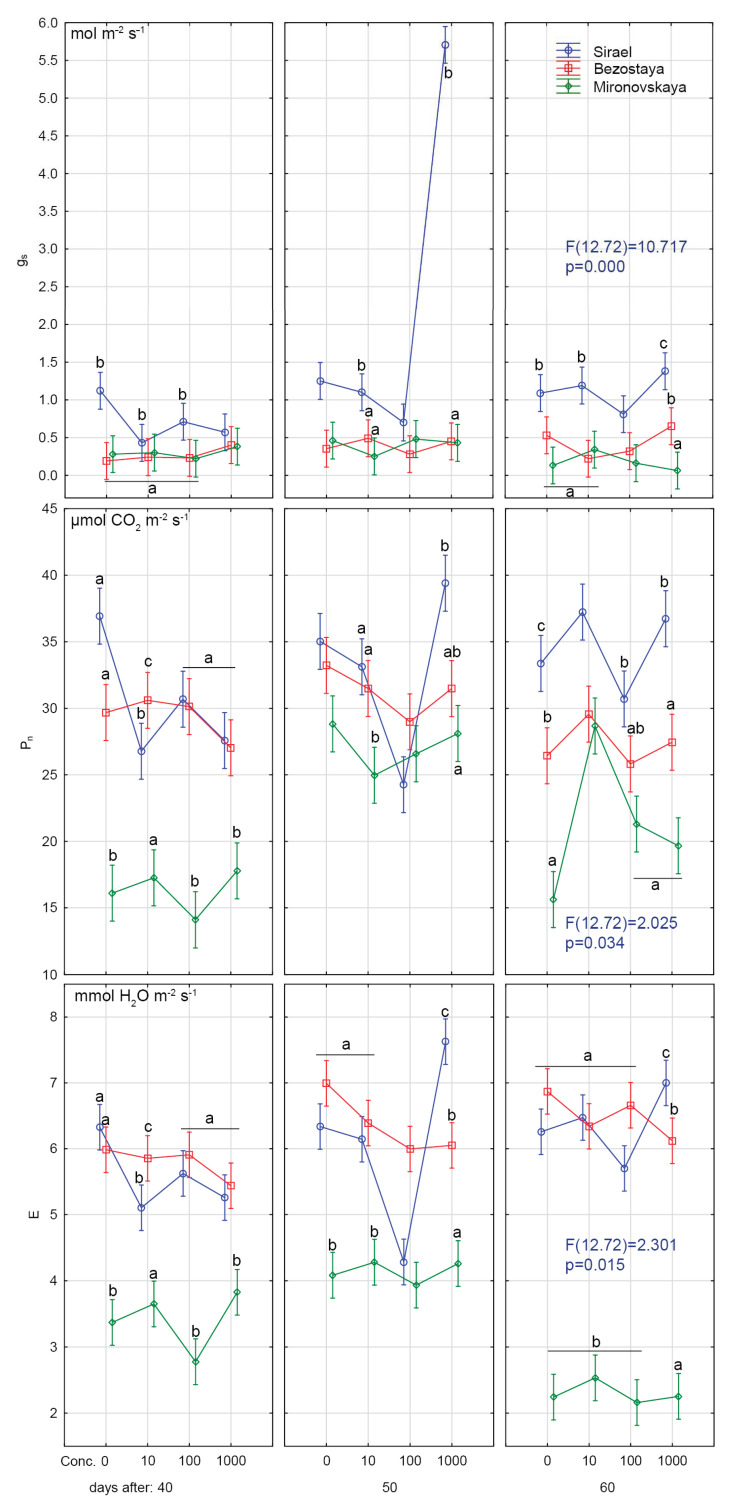
Effects of priming GA_3_ at different concentrations on photosynthetic rate (P_n_), stomatal conductance (g_s_) and transpiration (E). Variety: Mironovskaya (M), Bezostaya (B) and Sirael (S). Concentrations of GA_3_: 0, 10, 100 a 1000 µL L^−1^; measured on 40 days, 50 days and 60 days of growth. Tukey HSD test, *α* = 0.05. Treatments with the same sign did not differ significantly at *p* < 0.05.

**Figure 3:**
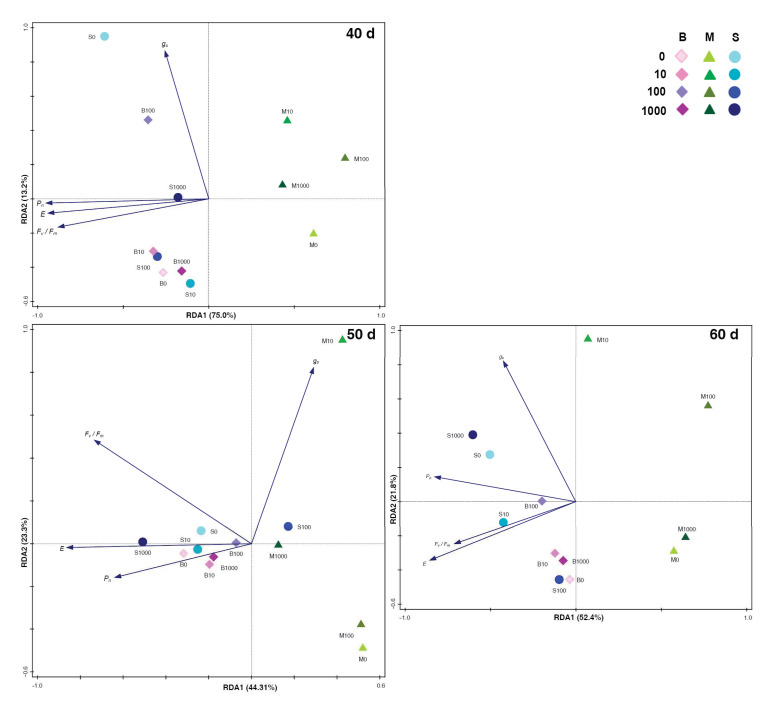
Ordination diagram (RDA—Redundancy analysis). The biplot displays the varieties with different GA_3_ concentrations. The explained variability is shown for the axes in the figures. The GA_3_ concentration is shown by the color intensity of the symbol that represents varieties (table in graph).

The theoretical maximum F_v_/F_m_ for C3 plants is about 0.83 [38]. Sharma et al. [39] obtained F_v_/F_m_ values of 0.79–0.84 for control wheat plants. In our experiments, the values of F_v_/F_m_ in the spring variety Sirael and the winter variety Bezostaya in all treatments and samplings ranged from 0.80 to 0.82 (Figure 1, Figure 2 and Figure 3). The measured values of F_v_/F_m_ for these varieties are similar to the average values of stress-free C3 plants.

### 2.1. Results at Forty Days of Growth

Statistically significant lower values of F_v_/F_m_ (0.53–0.61) were found for the winter variety, Mironovskaya, at all GA_3_ concentrations tested (Figure 1, Figure 2 and Figure 3). Lower values than optimal for C3 plants indicate the effect of adverse conditions such as water stress [40], heavy metals stress [41], and salt stress [42]. Sharma et al. [39] considered stress F_v_/F_m_ values in wheat to be 0.75–0.82 compared to stress values in corn plants, which were in the range of 0.31 to 0.64 [43]. Values under 0.6 indicate that the stress is affecting PSII [38]. Changes in the content of pigment, the F_v_/F_m_ ratio and non-photochemical quenching usually occur as a reaction to abiotic stress caused by high light levels [44]. From the experiments of [7], we see that long day irradiance can change the flowering time and cause the vegetative organs of wheat to develop insufficiently. This also results in lower values of F_v_/F_m_ through effects on PSII. Sirael and Bezostaya showed +/- identical values, indicating that PSII was functional. Mironovskaya (40 days sampling) showed very low values of F_v_/F_m_, which were probably caused by the experiment environmental condition. The application of GA_3_ led to a slight improvement of these parameters (statistically inconclusive). Which may be related to the fact that GA_3_ priming supported the formation of chlorophyl or slowed down its decomposition [39]. Thus, the Mironovskaya variety showed a statistically non-significant increase in F_v_/F_m_ with increasing GA_3_ concentration. These results confirm the findings of [45], which showed that the chlorophyll content gradually increased as GA_3_ concentration was increased, resulting in higher F_v_/F_m_ values. Increasing the concentration of GA_3_ could have a favorable effect on the content of chlorophyll and thus the improvement of F_v_/F_m_ parameters. The rate of P_n_, E and g_s_ was significantly lower in the Mironovskaya variety compared to other varieties, while GA_3_ treatments did not have a significant effect, similar to ryegrass [46].

### 2.2. Results at Fifty Days of Growth

Similar to the 40-day sampling results, the values of F_v_/F_m_ at 50 days in Bezostaya (0.79–0.81) and Sirael (0.80–0.82) treated with GA_3_ corresponded to the characteristic values for C3 plants under optimal conditions. For Mironovskaya, the F_v_/F_m_ values ranged from 0.71 to 0.8. Significantly, lower F_v_/F_m_ values were measured in the Mironovskaya variety at GA_3_ concentrations of 0 mg L^−1^, 10 mg L^−1^ and 100 mg L^−1^. The results show an improvement in the parameter F_v_/F_m_ in Mironovskaya, but a significant improvement in the Sirael and Bezostaya variety, which were in good condition (Figure 1, Figure 2 and Figure 3). Similarly, a trend towards improvement was indicated by P_n_, E, g_s_.

### 2.3. Results at Sixty Days of Growth

On this sampling date, no statistically significant differences between Mironovskaya, Sirael and Bezostaya in the values of F_v_/F_m_ were found for any of the varieties at any GA_3_ concentration tested. As stated above, the measured values and trends of F_v_/F_m_, P_n_, E and g_s_ of the spring variety Sirael and the winter variety Bezostaya were equal. On the last day of sampling, some measured physiological characteristics of PSII (P_n_, E and g_s_) in the Mironovskaya variety were increased to the level of other varieties in comparison with the first screening.

### 2.4. Development of the Growing Point

An overview of developmental stages of the individual wheat varieties at 40, 50 and 60 days of growth is shown in Table 1. The double-ridge stage is considered the moment of transition of the growing point from the vegetative to generative stage of development [47,48,49]. According to [50], the double ridge in wheat cannot be achieved without exposure to low temperatures. Flood and Halloran [51] indicated that, in general, spring wheats were either not sensitive or moderately sensitive to vernalization. On the other hand, winter wheats had a strong response to vernalization and required an interval of cold temperatures for the induction of flowering [52]. The vernalization requirement of the Mironovskaya variety is 42–49 days, whereas in the Bezostaya variety, it is 35–42 days [53]. Some varieties are photoperiod-sensitive only if the vernalization requirement is met [54]. Whitechurch et al. [55] showed that only 15% of the non-vernalized plants had reached anthesis, while the remaining plants continued to be vegetative until they senesced. According to [8], in some genotypes, the need for exposure to low temperature may be replaced by the effect of short days. Evans [56] specified that the vernalization requirement in winter wheats could be fully substituted by short days at non-vernalizing temperatures of 16–21 °C.

**Figure 4:**
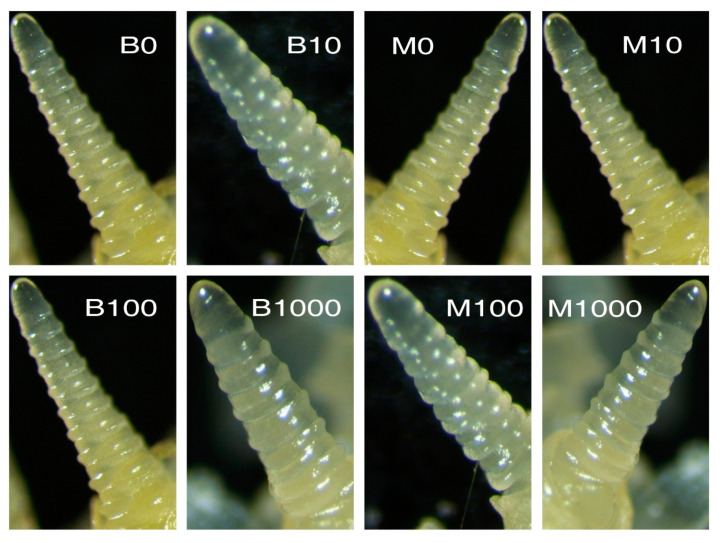
The growing point in various concentrations of GA_3_ in the varieties after 60 days of growth. Varieties: Bezostaya (B), left panel, and Mironovskaya (M), right panel. Concentrations of GA_3_: 0, 10, 100 a 1000 mg L^−1^; Phenological stage: prolonged single ridge.

**Figure 5:**
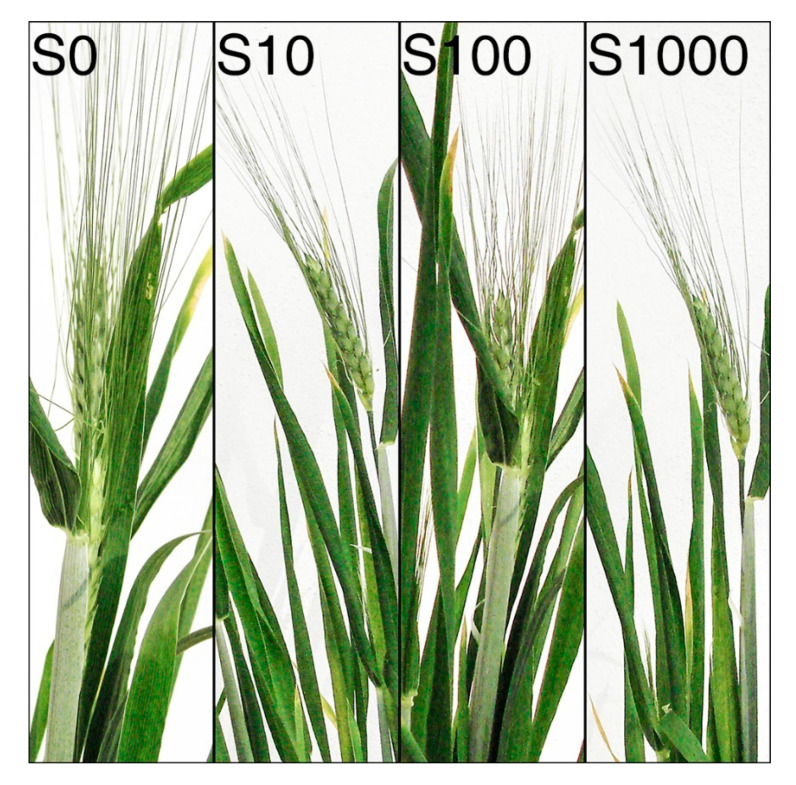
Development of heads in the spring variety after 60 days of growth. Variety: Sirael (S); Concentrations of GA_3_: 0, 10, 100 a 1000 mg L^−1^; Phenological stage: head emergence.

The results of our experiments revealed that after 60 days, the growing points in the winter wheats, Mironovskaya and Bezostaya (Figure 4), were at the single-ridge stage at all GA_3_ concentrations; therefore, the transition into the generative stage did not take place. The control spring type, Sirael, however, was already in the head emergence stage after 60 days at all GA_3_ concentrations tested (Figure 5). We conclude that the Sirael variety does not require vernalization or a short-day photoperiod to transition to the generative stage. According to [57], despite the occurrence of flowering, it is concluded that the role of GA in this phenomenon is restricted to the activation of lateral meristems in the apex. Mironovskaya achieved head-emergence on average a week later than Bezostaya with the same vernalization period of eight weeks [58]. Unless Mironovskaya is exposed to sufficient vernalization, its development is delayed or can even result in absence of head emergence. In high-stem winter wheat varieties, the application of GA_3_ did not significantly enhance the transition from vegetative to generative flowering stage because plants treated with GA_3_ flowered only one to two days earlier than controls [35]. In our experiments, the transition to the generative stage did not take place in the non-vernalized winter varieties Bezostaya and Mironovskaya, even after treatment with GA_3_. A one-time application of GA_3_ by seed soaking had no effect on the cancellation of the vernalization requirement, the transition from the vegetative to the generative stage in varieties affected the progress of spike development. This differs from the results of [49,59] after a one-time application of GA_3_ during the vegetative phase. These results could be explained according to [18], who stated that a new synthesis or activation of GAs takes place during the second stage of vernalization after the effect of lower temperatures. That would explain why it is not possible to substitute GA application for vernalization on plants in their embryonic state.

**Figure 6:**
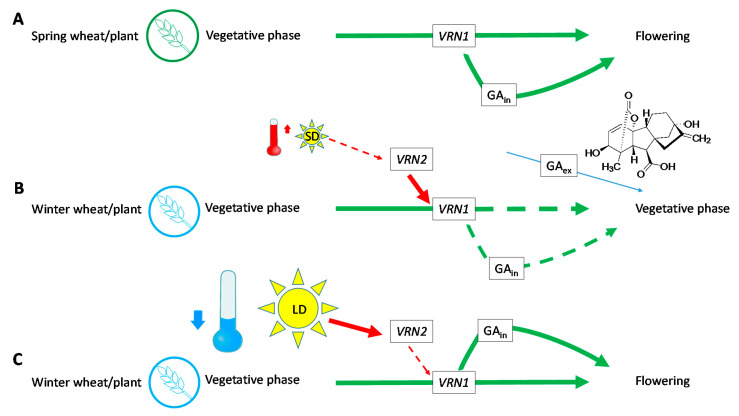
Scheme: activation of flowering within spring (**A**) and winter varieties without (**B**) or with vernalization (**C**) according to [5]. (SD—short day, LD—long day, GA_in_—native phytohormones, GA_ex_—applied GA_3_).

As stated by [60], spring wheat varieties are not dependent on activity of *VRN2*, whether by its damage, loss or changes in the regulatory part of *VRN1* and as result, they have no need of the vernalization process (Figure 6A). However, these plants still require the functional *VRN1* gene; without it, they remain in vegetative forms. According to [5], *VRN1* positively affects the changes in apex and production of GAs, when both processes are needed, and subsequently lead to spike development. *VRN1* fusion accelerates the reproductive development in transgenic barley plants [52]. In the case of winter wheat varieties, lack of vernalization (Figure 6B) does not inhibit *VRN2*, which suppresses *VRN1*, and the differentiation to flowering apex does not occur here. Production of native GAs could also be decreased [5] and exogenous applications of these hormones (blue arrow) on seeds did not substitute for vernalization, as was verified with used concentrations (Figure 4) in the plants grown under the specified conditions (Table 2).

Nevertheless, they still had some positive effect on elongation of *Phleum pratense* tillers under shorter days [61] and the authors further discussed the sensitivity of different cultivars to this phytohormone in non-vernalized plants under different conditions. Regarding plants from the *Poaceae* family, previous research showed that GA, also applied on leaves as in the case of [61], substituted the effect of longer days in *Lolium perenne* [62]. It also supported the rate of leaf extension, but it had no effect on photosynthetic parameters or specific leaf area [46]. Winter wheat varieties, which went through lower temperatures, have suppressed VRN2 and VRN1 and can start the flowering process (Figure 6C) [5].

## 3. Materials and Methods

### 3.1. Plant Material

Varieties of common wheat (*Triticum aestivum* L.) with different sensitivities to photoperiod and vernalization were selected. Varieties used included: Bezostaya 1 (B)—winter wheat (*vrn-A1, vrn-B1, vrn-D1*) with minor sensitivity to photoperiod (*Ppd-D1a*) and weak vernalization requirement (4 weeks, chilling requirement depends also on temperature; 1–3 °C); Mironovskaya 808 (M)—winter wheat (*vrn-A1, vrn-B1, vrn-D1*), sensitive to photoperiod (*Ppd-D1b*) and strong vernalization requirement (8 weeks, chilling requirement depends also on temperature; 1–3 °C); and Sirael (S)—spring wheat (*Vrn-A1a, Vrn-B1c, vrn-D1, vrn-B3*), sensitive to photoperiod (*Ppd-D1b*), but not sensitive to vernalization (average period of head emergence 67 days; used as control). Information regarding the genetic background of these varieties follow [53,58,63].

### 3.2. Gibberellin GA_3_ Treatment of Seeds

Seeds of each variety were treated by soaking with four concentrations of GA_3_ (0, 10, 100 and 1000 mg L^−1^ (10 seeds, *n* = 3). According to [64], soaking seeds has a more long-term effect than spraying and the chemical is also more evenly absorbed by the seeds [65]. The powder form of GA_3_ (Sigma, St. Louis, MO, USA, minimum 90% total gibberellins) is insoluble in water and 70% medical-grade ethanol was added to the weighed amount of GA_3_ to dissolve it. The GA_3_ solution was then diluted with distilled water to the required concentrations. The control seeds were soaked in distilled water only (0 mg L^−1^). The seeds were placed into 2 mL Eppendorf tubes and covered with 1 mL GA_3_ solution at the desired concentrations (28.9 μM; 289 μM or 2.89 mM), or 1 mL of distilled water as control, and held at 21 °C for 16 h.

### 3.3. Growing Conditions

After GA_3_ treatment, the caryopses were rinsed and transferred to Petri dishes containing filter paper moistened with distilled water and held for 36 h at a temperature of 22 °C. This method of germinating the caryopses followed the recommendation of the ISTA (International Seed Testing Association, Bassersdorf, Switzerland). After two days, non-viable caryopses were removed, and germinated caryopses were transplanted into 11 × 11 × 12 cm containers filled with growing substrate. The growing substrate was composed of enriched peat (pH 5.5–6.0, incinerable compounds min. 35%, particles over 25 mm max. 5%, overall N: 80–120 mg L^−1^, P_2_O_5_: 50–100 mg L^−1^, K_2_O: 100–150 mg L^−1^) and sand at a ratio of 3:1. Plants were transferred to an air-conditioned chamber (Conviron E8 + control unit CMP6050) and grown under a long-day photoperiod of 16 h for 60 days (Table 2). The plants were fertilized with a 5% solution of NPK (15-5-5) (GSH NPK 15-5-5, Lovochemie a.s., Lovosice, Czechia) once every 14 days and with the microelement solution according to Benson once per month.

### 3.4. Photosynthesis Measurement

Measurements of potential photochemical effectiveness of electron transport as given by F_v_/F_m_, were performed on the plants and the stage of development of plants was established using micro-phenological degrees of the growing point [66]. These measurements were performed at 40, 50 and 60 days of growth. Given the possible differences of the developmental stages between the individual modifications, the time interval for sampling was selected regardless of their phenological stage of development. The phenological stage and uniformity of development of the individual varieties was controlled by sampling their growing points [66]. The chlorophyll fluorescence parameters—minimum (F0) and maximum (Fm) were measured on two fully developed intact leaves of each variety on three different plants by the portable ADC:OSI FL 1 analyzer (ADC BioScientific Ltd., Hoddesdon, UK) with 1 s excitation pulse (660 nm) and saturation intensity 8000 μmol m^−2^ s^−1^ after 20 min dark-adaptation of the leaves. The maximal quantum efficiency of PSII was calculated as F_v_/F_m_ = (F_m_ − F_0_)/F_m_ and the efficiency of the water-splitting complex on the donor side of PSII (as inferred from F_v_/F_0_) [67]. Leaf gas exchange parameters—the net photosynthetic rate (P_n_), rate of transpiration (E) and stomatal conductance (g_s_)—were measured at same time as the chlorophyll fluorescence parameters, using the portable gas exchange system LCpro+ (ADC BioScientific Ltd., Hoddesdon, UK).

### 3.5. Identifying the Microphenological Stage of the Growing Point

The stage of phenological development was established according to the changes of the growing point. The stage was identified using the method of [47,66] for spring wheat. Three plants from each variety and each modification were sampled and their growing points extracted. The average phenological stage was established based on these points. The growing points were imaged with a stereoscopic microscope (Nikon SMZ645, Nikon, Japan) and the data were processed using NIS-Elements AR 4.5 software.

### 3.6. Statistical Analysis

One-way ANOVA was used to evaluate the effect of concentration of GA_3_ on the varieties. It was always compared at a single concentration. After obtaining significant results (*p* < 0.005), multiple comparisons using Tukey HSD test were applied to identify significant differences between treatments. Correlation coefficient was used for interspecies interaction. All analyses were performed using STATISTICA 13.5 (Statsoft, Tulsa, OK, USA). Canoco 5 [68] was used for RDA (redundancy analysis) calculated from centered (but not standardized) data. This analysis was appropriate for finding the difference between varieties (M, B, S) with different concentrations.

## 4. Conclusions

The development of the growing point in winter varieties occurred during the prolonged single-ridge stage. In the control, the spring variety reached the stage of head emergence after 60 days of growth. A one-time application of GA_3_ through seed soaking had no effect on the transition of the growing point to the generative double-ridge stage.

### Perspectives

The main fundamental issues of vernalization are how plants sense the signal of vernalization and calculate the dose of long-term cold exposure, along with how these processes can be applied to crop production. The winter wheat variety, Mironovskaya, is very sensitive to the photoperiod. This means that during flowering it should be exposed to a long day (14 + h); before that, it must be exposed to low temperatures for several weeks to promote vernalization. Application of GA_3_ on seeds did not replace the requirement for vernalization. The winter wheat variety, Bezostaya, is less sensitive to photoperiod (low temperatures for four weeks), and the application of GA_3_ also did not replace the requirement for vernalization. The spring variety, Sirael, is sensitive to photoperiods and very little to vernalization. Further research could include testing of GA_3_ application to short-straw winter wheat varieties, which could promote the prolonged growth and transition to the flowering phase and replace the requirement for vernalization.

## Figures and Tables

**Table 1:** Overview of developmental stages of wheat varieties at 40, 50 and 60 days of growth.

Days After	40				50				60			
Variety/Conc.	0	10	100	1000	0	10	100	1000	0	10	100	1000
Mironovskaya	EE	EE	EE	EE	SR	SR	SR	SR	PSR	PRS	PSR	PSR
Bezostaya	EE	EE	EE	EE	SR	SR	SR	SR	PSR	PSR	PSR	PSR
Sirael	2 ND	2 ND	2 ND	2 ND	SE	SE	SE	SE	H	H	H	H

EE, early elongation, SR, single ridge, PSR, prolonged single ridge, 2 ND, second node detectable, SE, stem elongation, H, heading.

**Table 2:** Growing conditions in the air-conditioned chamber.

Time (16 h day/8 h night)	Temperature °C	Relative Humidity Φ (%)	Photosynthetic Photon Flux Density (PPDF) µmol m^−2^ s^−1^
Dawn (5:00–6:00)	17	65	280
Day (6:00–20:00)	20	60	560
Nightfall (20:00–21:00)	17	65	280
Night (21:00–5:00)	15	70	0
